# Disassembly of Dimeric Cyanine Dye Supramolecular Assembly by Tetramolecular G-quadruplex Dependence on Linker Length and Layers of G-quartet

**DOI:** 10.3390/molecules24102015

**Published:** 2019-05-27

**Authors:** Lijia Yu, Yansong Zhang, Chunguang Ding, Xiaodong Shi

**Affiliations:** National Center for Occupational Safety and Health, National Health Commission of the People’s Republic of China, Beijing, China; zyszhys@163.com (Y.Z.); 13466322180@163.com (C.D.); xdshi@noon.ibp.ac.cn (X.S.)

**Keywords:** dimeric cyanine dye, tetramolecular G-quadruplex, assembly and disassembly

## Abstract

Cyanine dyes have been widely applied in various biological systems owing to their specific photochemical properties. Assembly and disassembly process of cyanine dyes were constructed and regulated by special biomolecules. In this paper, dimeric cyanine dyes with different repeat units (oligo-oxyethylene) in linker (TC-Pn) (*n* = 3–6) were found to form H-aggregates or mixture aggregates in PBS. These aggregates could be disassembled into dimer and/or monomer by (TGnT) tetramolecular G-quadruplexes (*n* = 3–6, 8), which were affected by the linker length of dimeric cyanine dyes and layers of G-quartets. The ^1^H-NMR titration results suggest that the binding mode of dimeric cyanine dye with TGnT might be on both ends—stacking like a clip. This binding mode could clearly explain that matching structures between dimeric cyanine dyes and TGnT quadruplexes could regulate the disassembly properties of aggregates. These results could provide clues for the development of highly specific G-quadruplex probes.

## 1. Introduction

The G rich oligomers form four stranded structures maintained by stacked G-quartets. The building blocks of G-quadruplexes are structures known as G-quartets (Gq), which are planar association of four G bases held together by eight Hoogsteen hydrogen bonds. The G-quartets are stacked on top of each other by hydrophobic interactions, stabilized by monovalent cations, such as K^+^ and Na^+^ [1]. G-quadruplex motifs are widespread in the telomere and promoter regions of several important oncogenes, and play an important function in cancer cell proliferation [2,3,4,5,6,7,8]. In addition, intermolecular G-quadruplexes also play important roles in the assembly and regulation of nucleic acid nanostructures (nanowires, strips, nanotubes) [9,10,11]. Tetramolecular G-quadruplexes are one of the important intermolecular G-quadruplexes, which are formed by four parallel strands bound together by n layers of G-quartets [12]. It is well known that the binding mode between tetramolecular G-quadruplex and ligands mainly are the groove-embedding mode and end-stacking binding mode [13,14,15,16]. A clip-like binding mode of dimeric cyanine dye with parallel intramolecular G-quadruplexes was found by our previous study [17,18]. This binding mode involves two binding modes of terminal stacking and groove-embedding. Thus, this clip-like binding mode of cyanine dye could improve the specific recognition of G-quadruplexes. 

**Figure 1:**
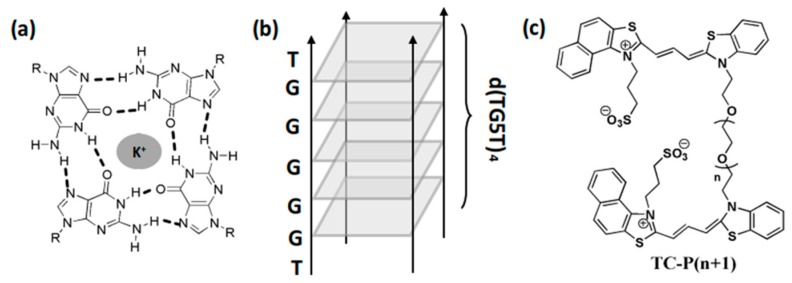
(**a**) Schematic representation of the G-quartet, (**b**) d(TG5T)_4_ (abbreviated TG5T) tetramolecular G-quadruplex formed by self-association of four parallel DNA, and (**c**) structures of TC-P(n + 1) (*n* = 2–5).

Cyanine dyes were widely applied in various biological systems due to their controllable process [19,20]. Assembly and disassembly processes of cyanine dyes can be constructed and regulated by biomolecules [21,22,23]. A cyanine dye named DMSB can be assembled into J-aggregates when biomolecular TBA G-quadruplex is added [24]. It can also bind to the intermolecular parallel G-quadruplex [d(TGGGGT)]_4_ (abbreviated TG4T) in the form of a dimer and monomer [25]. The high specificity of DMSB makes it an excellent G-quadruplex structural probe. A series of dimeric cyanine dyes (Figure 1c) were designed in our previous work [17]; it consisted of two monomeric parent cores bridged by a linker chain. The monomeric part of dimeric cyanine dye was similar to DMSB. The major difference in the monomeric part of dimeric cyanine is replacement of one side of the benzoselenazole scaffold with the naphthothiazole unit, as well as addition of the cationic sulfo group to the N-alkyl chain. Owing to this structural change, we speculate that assembly and disassembly of dimeric cyanine dye could be affected by linker length. In addition, the clip-like binding mode of dimeric cyanine dyes could also be regulated by the layers of G-quartets.

In this paper, the interaction between dimeric cyanine dyes with various linker length (TC-P3-6) and TGnT (*n* = 3–6, 8) G-quadruplex with n layer of G quartets was studied (Figure 1). Dimeric cyanine dyes formed H-aggregates or mixture aggregates in PBS buffers. The disassembly property of the same dimeric cyanine dye was greatly affected by layers of d(TGnT)_4_ (abbreviated TGnT) G-quadruplexes. Same TGnT G-quadruplex showed the capability for various disassembly aggregates of dimeric cyanine dyes (TC-P3, TC-P4 and TC-P5). The results of ^1^H-NMR titration of TG4T G-quadruplex with TC-P4 cyanine dye show that the binding mode should be clip-like, as our study posits. 

## 2. Result and Discussion

### 2.1. Spectral Properties of Dimeric Cyanine Dyes in DMSO and PBS

**Figure 2:**
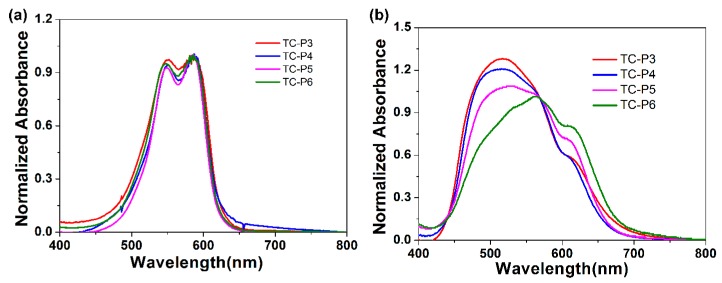
Absorption spectra of 10 μM of various cyanine dyes: (**a**) TC-P3-6 in DMSO, (**b**) TC-P3-6 in PBS buffer.

Cyanine dye structure and environment play an important function in the assembly and disassembly of dyes [26]. Some cyanine dyes are able to form H-aggregates or J-aggregates in special environments [27,28]. As shown in Figure 2, dimeric cyanine dyes (TC-P3-6) can form a mixture of dimer (550 nm) and monomer (586 nm) in DMSO, and mixture aggregates or H-aggregates (492 nm) in PBS with similar compounds [17,29,30] and an exciton model [31]. These aggregates of dimeric cyanine dyes can be regulated by different DNA motifs.

### 2.2. The Interaction Between Dimeric Cyanine Dyes and TGnT G-quadruplex

**Figure 3:**
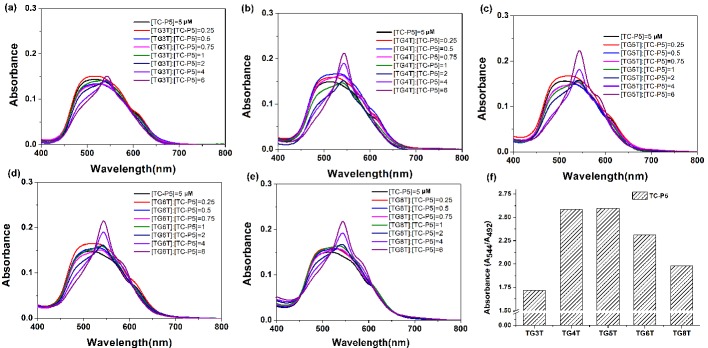
(**a**–**e**) Absorption spectra of 5 μmol/L TC-P5 at various concentrations of TGnT (*n* = 3–6, 8) G-quadruplexes. All spectra data of the (**a**–**e**) are normalized to absorbance at 800 nm (A_800_ = 0). (**f**) The ratio value of absorbance at 594 nm via absorbance at 492 nm at a function of [TGnT]/[TC-P5] = 6 (*n* = 3–6, 8).

The binding mode of dimeric cyanine dye with parallel G-quadruplex c-myc was on both ends of the G-quadruplex like a clip. This binding mode is greatly affected by the linker of the dimeric cyanine dye and layers of the G-quadruplex. Here, we investigate the interaction between dimeric cyanine dyes with different linker lengths and tetramolecular G-quadruplex with various layers of G-quartets. The d[TGnT]_4_ (abbreviated TGnT) tetramolecular quadruplexes, readily identifiable by its NMR spectrum, is considered as the thermodynamically stable strand assembly [32,33]. The absorption titration spectra of TGnT (*n* = 3–6, 8) G-quadruplexes with various layer G-quartets (Table S1 and Figure S1, supporting information) with dimeric cyanine dyes TC-P5 was investigated. As shown in Figure 3, TC-P5 exhibits distinct spectral changes with the addition of various TGnT tetramolecular G-quadruplexes, although the only difference in their structure is the layers of G-quartets. TG3T G-quadruplex could hardly disassemble TC-P5 aggregates at 492 nm (H-aggregates), even at a high concentration of 30 μM. TG4T, TG5T, TG6T and TG8T were all able to cause a great increase of TC-P5 dimer at 544 nm and a small shoulder band of monomer at 592 nm, accompanied with significant falling off of H-aggregates at 492 nm. This result suggests that TC-P5 interacts with TGnT (*n* = 4–6, 8) in the form of dimer and monomer. It indicates that TGnT is able to induce TC-P5 H-aggregates into dimer and monomer when TGnT (*n* = 4–6, 8) is in excess. Since TGnT tetramolecular G-quadruplex disassembles TC-P5 aggregates differently, the absorption value of H-aggregates at 492 nm (A_492_) could be estimated as a disassembly ability of TC-P5. The absorption value at 544 nm assigned to dimer. The value of A_544_/A_492_ was used to reflect the disassembly H-aggregates into dimer ability. As shown in Figure 3f, the order value of A_544_/A_492_ for TC-P5 with TGnT (*n* = 3–6, 8) followed TG5T > TG4T > TG6T > TG8T > TG3T at a ratio of [TGnT]/[TC-P5] = 6, indicating that TG4T and TG5T were in favor of disassembly of H-aggregates of TC-P5 over that of TG3T, TG6T and TG8T tetramolecular G-quadruplexes (Figure S3, supporting information).

**Figure 4:**
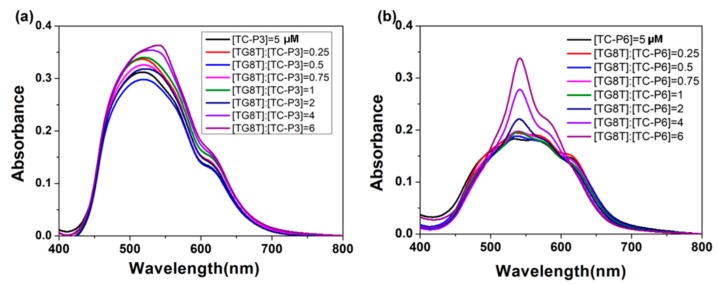
(**a**) Absorption titration spectra of 5 μmol/L TC-P3 at different concentrations of TG8T G-quadruplex; (**b**) absorption titration spectra of 5 μmol/L TC-P6 at various concentrations of TG8T G-quadruplex.

In order to clarify the linker length of dimeric cyanine dye’s influence on interaction with tetramolecular G-quadruplex, TG8T G-quadruplex with an eight-layer G-quartet was chosen to help TC-P3 interact with a shorter linker length and TC-P6 with a longer linker length. As shown in Figure 4, TG8T could not disassemble TC-P3 mixture aggregates, while TG8T G-quadruplex could induce TC-P6 aggregates into dimer (542 nm) and monomer (580 nm). These results show that TC-P6 might interact with TG8T G-quadruplex in the form of dimer and monomer. However, TC-P3 with a shorter linker length could not interact with TG8T in the dimer and/or monomer. 

**Figure 5:**
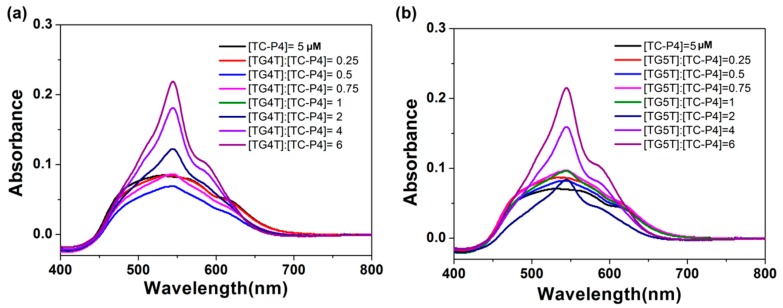
Absorption titration spectra of 5 μmol/L TC-P4 at distinct concentrations of TG4T (**a**) and TG5T (**b**) G-quadruplexes.

We have further chosen TC-P4 with a moderate linker length to interact with TG4T and TG5T tetramolecular G-quadruplexes. As shown in Figure 5, TG4T tetramolecular G-quadruplex could disassemble TC-P4 H-aggregates (492 nm) into dimer and monomer; similarly, TG5T tetramolecular G-quadruplex could also disassemble TC-P4 H-aggregates into dimer (542 nm) and monomer (580 nm). This result proved that TC-P4 with moderate linker length interacted with TG4T and TG5T with proper layers of G-quartets in the form of dimer and monomer mode. 

### 2.3. The Binding Mode of TC-P4 with TG4T

**Figure 6:**
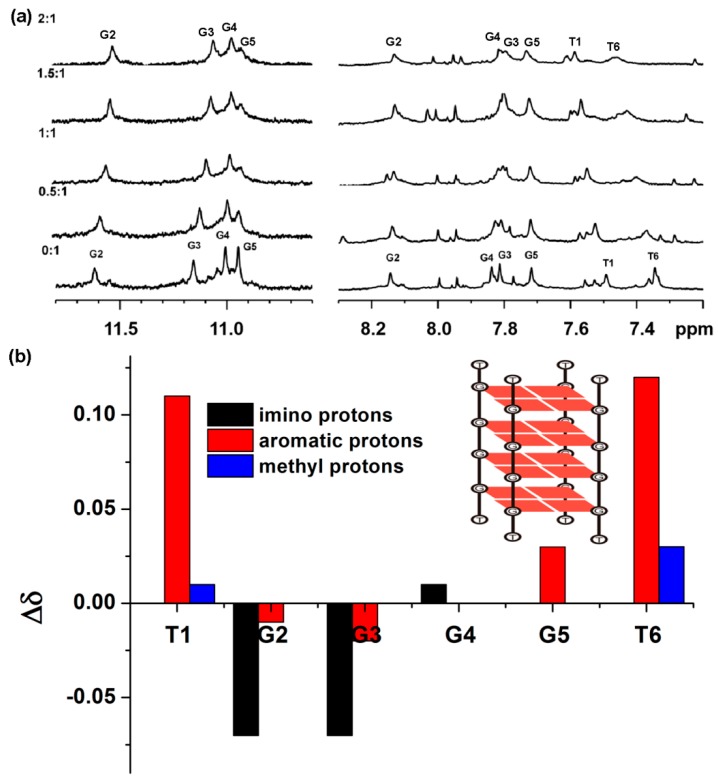
(**a**) The unambiguously assigned ^1^H-NMR titration spectra of 120 μM TG4T with different concentrations of TC-P4 in 0.6 mL PBS (10 mM KH_2_PO_4_, 70 mM KCl, 1 mM EDTA, pH 7.4 H_2_O/D_2_O, 9/1, *v*/*v*); (**b**) schematic topology diagram for TG4T G-quadruplex. Difference in chemical shifts of base protons on TG4T with interaction of TC-P4. Values are reported for aromatic (red), imino (black) and methyl (blue) protons.

In order to clarify binding sites of dimeric cyanine dye with tetramolecular G-quadruplex, ^1^H-NMR titration between TC-P4 and TG4T G-quadruplex was carried out. 3D molecular structure of the TG4T has been determined by NMR and X-ray techniques; the strands associate with generating a right-handed helix, containing four equivalent grooves and all bases in the anti glycosidic conformation [14,22]. In Figure 6 and Figure S2 (supporting information), ^1^H-NMR titration spectra of 0.12 mM TG4T with various concentrations of TC-P4 in PBS at 308 K is shown. The TG4T resonance signals for four imino protons (10–12 ppm), six aromatic protons (7–9 ppm), and two thymine methyl protons (1–2 ppm) were well resolved. Clearly, the resonance signals for G5-NH and T6-H6 gradually disappear. T6-H6 and T1-H6 show a remarkable downfield shift. The changes of the chemical shifts of T1 and T6 protons (>0.1 ppm) are much larger than those of others, suggesting that the binding site for TC-P4 with TG4T is probably located on T1 and T6. T6 is 3′-terminal G-quartet and T1 is 5′-terminal G-quartet. It is reasonable that TC-P4 could stack on T1 and T6 through both end-stacking modes. Based on this binding mode, TC-P5 and TC-P4 both have a better matching length to interact with four and five G-tetrads of TGnT tetramolecular G-quadruplex. TC-P3 with a short linker length could not bind to TG8T in the form of monomer like a clip due to its short linker length, while TC-P6 aggregates could be induced into dimer and monomer due to a matching linker length. 

## 3. Materials and Methods 

### 3.1. Synthesis of Dimeric Cyanine Dyes

The synthesis of TC-P3-P6 has been described in our previous study [17]. All these structures were verified by nuclear magnetic resonance and mass spectrometry. 

### 3.2. Preparing of Samples

All these oligonucleotides of [d(TGGGT)]_4_ (TG3T), [d(TGGGGT)]_4_ (TG4T), [d(TGGGGGT)]_4_ (TG5T), [d(TGGGGGGT)]_4_ (TG6T) and[d(TGGGGGGGGT)]_4_ (TG8T) were purchased from Sangon Biotech Co., Ltd. (Shanghai, China) and purified by HPLC. Ultrapure water was prepared by a Milli-Q gradient ultrapure water system (Millipore, Molsheim, France). 

The stock solution of 100 μM dimeric cyanine dyes was prepared by dissolving into DMSO and storing in the dark at room temperature. PBS buffer (10 mM KH_2_PO_4_-K_2_HPO_4_, 70 mM KCL, 1 mM EDTA, pH 7.4) was used to dissolve the oligonucleotides. The absorbance at 260 nm was used to determine the concentrations of oligonucleotides. All oligonucleotides were heated to 95 °C for 5 min, rapidly cooled to 4 °C and kept overnight at 4 °C. Their folding topologies were identified by circular dichroism (CD) before usage. 

The measured samples were prepared by corresponding DNA stocking solutions into 100 μL PBS containing 5 μM dyes. The final volume was 700 μL after addition of PBS. The samples were incubated for 12 h in the dark at room temperature before measurements we taken. 

### 3.3. Spectral Measurements

The UV-vis absorption spectra were recorded using an Agilent-8453 spectrophotometer (Santa Clara, CA, USA) equipped with a Peltier effect heated cuvette holder in a 10 mm quartz cell. All the CD spectra were recorded on a JASCO J-815 spectrophotometer (Tokyo, Japan) in a 10 mm quartz cell at room temperature. All CD spectra were collected with a scan speed of 500 nm/min and a response time of 0.5 s between 200 nm and 350 nm with three scans averaged. 

The ^1^H-NMR titration spectra were carried out by a Bruker Avance 600 spectrometer (Fällanden, Switzerland) equipped with a BBI probe. The stocking solution of TG4T was prepared by dissolving in PBS [10 mM K_2_PO_4_/KH_2_PO_4_, 70 mM KCl, 10% D_2_O/90% H_2_O (*v*/*v*)]. The spectra was recorded immediately by addition of TC-P4 dissolved in DMSO-*d6*. Bruker pulse program p3919 gp was used to suppress water peak. 

## 4. Conclusions

In this study, the spectra characteristics of dimeric cyanine dye with tetramolecular G-quadruplex were investigated. In PBS, dimeric cyanine dyes could form H-aggregates or mixture aggregates. These aggregates could be transformed into dimer and/or monomer by tetramolecular G-quadruplex with proper layers of G-quartets. ^1^H-NMR titration results provide binding sites of dimeric cyanine dyes location at both ends of tetramolecular G-quadruplex. This work enriches our understanding on designing specific ligands of recognition of G-quadruplex and highlights the application potential of these G-quadruplex structures in regulation of supramolecular assembly of cyanine dye. 

## Supplementary Materials

The following are available online, Figure S1: The CD spectra for TG3T, TG4T, TG5T, TG6T and TG8T in 10 mM PBS (K^+^). Figure S2: The unambiguously assigned ^1^H-NMR titration spectra of 120 μM TG4T with different concentrations of TC-P4 in 0.6 mL PBS (10 mM KH_2_PO_4_, 70 mM KCl, 1 mM EDTA, pH 7.4 H_2_O/D_2_O, 9/1, *v*/*v*) in methyl protons. Figure S3. The ratio value of absorbance at 594 nm via absorbance at 492 nm at a function of [TGnT]/[TC-P5] = 4 (*n* = 3–6, 8). The concentration of TC-P5 is 5 μmol/L. Table S1: Sequences of 5 oligonucleotides.
