# Supplementary material for: Disassembly of Dimeric Cyanine Dye Supramolecular Assembly by Tetramolecular G-quadruplex Dependence on Linker Length and Layers of G-quartet
Source: Molecules. 2019 May 27;24(10):2015. doi: 10.3390/molecules24102015 (PMC6571858; doi:10.3390/molecules24102015)
Supplement: Supplementary file 1 [file molecules-24-02015-s001.pdf]

Supporting information for: Disassembly of Dimeric Cyanine Dye Supramolecular Assembly by Tetramolecular G-quadruplex Dependence on Linker Length and Layers of G-quartet

Lijia Yu [1,*], Yansong Zhang [1] , Chunguang Ding[1] and Xiaodong Shi [1]

[1] National Center for Occupational Safety and Health, National Health Commission of the People's Republic of China, Beijing, China ; yulijiajiayou@163.com(L.Y.)

* Correspondence: yulijiajiayou@163.com (L.Y.); Tel.: +0086-010-5615-3680 (L.Y.)

1. The sequences of 5 oligonucleotides in the study

Table S1. Sequences of 5 oligonucleotides

Name	Sequence[a] (from 5 to 3)	Motifs
TG3T	TGGGT	Parallel DNA G-quadruplex
TG4T	TGGGGT	Parallel DNA G-quadruplex
TG5T	TGGGGGT	Parallel DNA G-quadruplex
TG6T	TGGGGGGT	Parallel DNA G-quadruplex
TG8T	TGGGGGGGGT	Parallel DNA G-quadruplex

[a]These oligonucleotides were dissolved in PBS (K^+) (10 mM KH_2PO_4/K_2HPO_4, 70 mM KCl, 1 mM EDTA, pH 7.4)

2. The Structure identification of 5 sequences by CD spectroscopy.

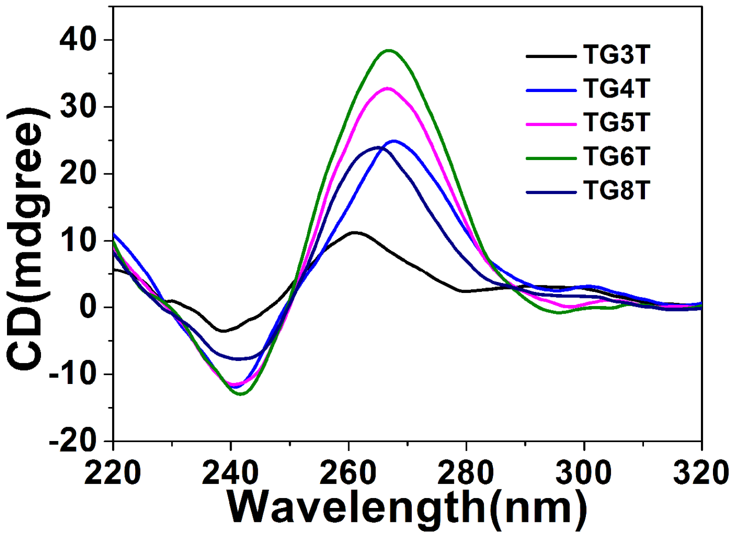

Figure S1. The CD spectra for TG3T,TG4T,TG5T,TG6T and TG8T in 10 mM PBS (K^+).

2. ^{1}H-NMR titration of TC-P4 with TG4T in methyl protons.

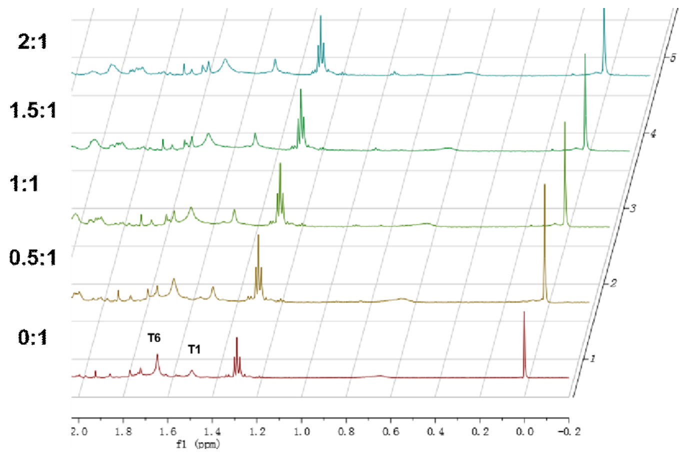

Figure S2. The unambiguously assigned ^{1}H-NMR titration spectra of 120 μM TG4T with different concentrations of TC-P4 in 0.6 mL PBS (10 mM KH_2PO_4, 70 mM KCl, 1 mM EDTA, pH 7.4 H_2O/D_2O, 9/1,v/v) in methyl protons.

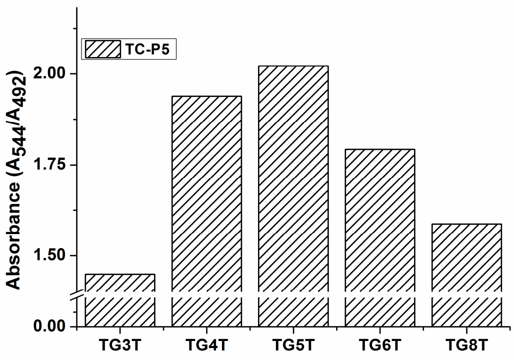

Figure S3. The ratio value of absorbance at 594 nm via absorbance at 492 nm at a function of [TGnT]/[TC-P5]=4 (n=3-6,8).The concentration of TC-P5 is 5 μmol/L.
